# Treatment with Minocycline Suppresses Microglia Activation and Reverses Neural Stem Cells Loss after Simulated Microgravity

**DOI:** 10.1155/2020/7348745

**Published:** 2020-04-25

**Authors:** Tian Lin, Juan Du, Li Liu, Zheng Wu, Xiangkai Kong, Yu Liu, Yiling Cai

**Affiliations:** Department of Neurology, The 306th Hospital of PLA, Beijing 100101, China

## Abstract

The present study aimed to investigate the effect of microglia on simulated microgravity-induced hippocampal neurogenesis reduction and the possible mechanism underlying. Adult rats were treated with tail suspension for different times and the changes of neural stem cells (NSCs) were examined by immunohistochemistry. Then, minocycline was used to inhibit the activation of microglia, and the numbers of microglia and NSCs were detected after microgravity. Additionally, liquid protein chip analysis was applied to detect proinflammatory factors in hippocampus in order to find out the cytokines responsible for microglia activation after microgravity. The results revealed that microgravity increased the numbers of Iba1^+^ cells and decreased the numbers of BrdU^+^ and DCX^+^ cells in hippocampus but did not affect the ratio of NeuN^+^/BrdU^+^ cells to the total number of BrdU^+^ cells. After treated with minocycline, activated microglia were suppressed and the reduction of NSCs induced by microgravity recovered. Besides, compared with the control, higher concentrations of INF-*γ* and TNF-*α* were detected in the rats treated with microgravity. Our study provides the first evidence that microglia-mediated inflammation plays an important part in microgravity-induced neurogenesis reduction in hippocampus, and INF-*γ* and TNF-*α* secreted by microglia might be the key factors in this process.

## 1. Introduction

With the extent of space exploration, the health and safety of space explorers exposed to a microgravity environment is drawing more and more attention. When entering into a microgravity environment, which is highly different from the Earth environment, astronauts undergo a series of physical changes. Many studies about microgravity have found that it can do harm to many body systems, especially the central nervous system (CNS), due to the specific structure and function of the brain [1, 2]. Previous research has shown that microgravity leads to learning and memory impairment in animals [3] and human being [4]. However, the mechanism of the effects remains unclear.

Functionally, learning and memory ability in adult mammals has great relation to the neurogenesis of the dentate gyrus in the brain, where granule neurons are generated throughout life from a population of neural stem cells (NSCs) [5]. NSCs in the subgranular zone (SGZ) of the dentate gyrus differentiate into newborn neurons, integrate into the existing circuitry, and receive functional input [6]. A group of studies have demonstrated that spatial learning [7] and memory [8] are facilitated in animals having more newborn neurons. Therefore, we wonder whether the hippocampal neurogenesis decreases after exposure to microgravity, resulting in the impairment of learning and memory ability. To test this hypothesis, in the present study, we exposed adult rats to microgravity and observed the changes in proliferation, migration, and differentiation of NSCs and tried to uncover the possible underlying mechanism.

The regulation of hippocampal neurogenesis is complicated and is usually affected by both intracellular and extracellular factors simultaneously [9]. Among numerous extracellular negative regulators, inflammation induced by microglia plays an important part. Detrimental effects of activated microglia on the survival of newly formed hippocampal neurons have been observed in vivo [10] as well as in vitro when NSCs were grown in conditioned media from lipopolysaccharides- (LPS-) activated microglial cells [11]. The key mechanism by which these adverse effects of microglia on neurogenesis are brought about is the release of proinflammatory mediators such as interleukin-1*β* (IL-1*β*), interleukin-6 (IL-6), interferon-*γ* (IFN-*γ*), tumor necrosis factor-*α* (TNF-*α*), and interleukin-18 (IL-18) from activated glia, which are antineurogenic [12–14].

As microglia are activated and their numbers are increased significantly in the spinal cord after exposure to microgravity [15], we wonder whether the activated microglia can have some adverse effects on NSCs. To test this possibility, the present study investigated the effect of microglia on neurogenesis in the dentate gyrus after exposure to microgravity and the possible process of function.

## 2. Materials and Methods

### 2.1. Animals

Adult male Sprague-Dawley rats (180–220 g) were locally bred under a 12/12 h light/dark cycle with ad libitum access to standard laboratory chow and water. To avoid any effect of unfavorable factors, including fear and stress, the rats were acclimatized to the rearing environment for seven days before the beginning of the experiments. The tail suspension (TS) rat model was used to simulate microgravity-induced alterations including the redistribution of body fluid and volume changes in cerebrospinal fluid. The TS rat model was first introduced by Morey-Holton and was later improved by Morey-Holton and Globus [16]. Briefly, the rats underwent an acclimation period during which they were tail-suspended for short durations (three days; 1–3 h per day) by either tail harness or stainless steel rings. For suspension, the tails were cleaned and dried, and adhesive sponge tape strips were adhered laterally along the two sides of the proximal two-thirds of the tail. These longitudinal strips were then secured to the tail by three 1 cm wide tape strips wrapped circumferentially at three sites along the length of the tail. To facilitate free movement about the cage, the cast was attached to a swivel anchored to the cage top, allowing a 360° range of movement. The rats were caged individually and maintained in a head-down tilt position of approximately 30° with their hind limbs unloaded. The control rats in individual cages were treated identically except for the TS.

### 2.2. Study Design

**Figure 1:**
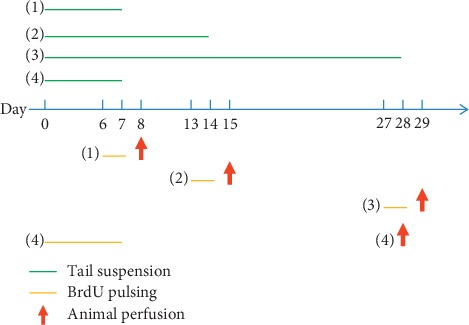
Experimental procedure and time course of tail suspension, BrdU administration, and animal perfusion. The rats in the tail suspension (TS) groups received tail suspension for 7, 14, or 28 d (indicated by green lines in (1), (2), and (3), respectively) and were sacrificed for proliferation assays. Twenty-four hours before killing (indicated by the arrow in (1), (2), and (3), respectively), these rats received three pulses of BrdU every 8 h for 24 h (indicated by yellow lines in (1), (2), and (3), respectively). The rats used for differentiation assays first received BrdU (100 mg/kg) injection each day for 7 d (indicated by yellow line in (4)) and then were perfused at day 21 (indicated by the arrow in (4)) after treatment with control or tail suspension.

Experiment 1 sought to examine the proliferation, migration, and differentiation of NSCs in the SGZ after exposure to microgravity. Animal grouping and drug injection are described in Figure 1. Briefly, 48 adult rats were randomly divided into a control group and a TS group. Then, rats of each group were equally divided into four subgroups, including three subgroups to observe the proliferation and migration of NSCs and one subgroup to evaluate the differentiation of NSCs. There were six rats in each subgroup. For proliferation and migration assay, the rats received 7 d, 14 d, and 28 d tail suspension, respectively, and were sacrificed at different times. The other rats received tail suspension for 7 d and were killed 21 d later.

Experiment 2 sought to investigate whether the inhibition of microglia activation affects the proliferation of hippocampal NSCs after exposure to microgravity. The tetracycline derivative minocycline (MC, Sigma-Aldrich) was administered systemically to inhibit microglia activation specifically [17]. A total of 24 rats were randomly divided into control, MC, TS, and TS + MC groups. A total of six rats were included in each group. Rats from the latter two groups received TS. Rats in the MC and TS + MC group received 50 mg/kg of minocycline once daily for the seven days of TS. The other rats received an equal injection of saline. Based on experiment 1, all rats were sacrificed after the seven-day tail suspension for proliferation assay.

Experiment 3 sought to study further whether the concentration of proinflammatory mediators changed after exposure to microgravity. A total of 36 rats were randomly divided into control group (*n* = 18) and TS group (*n* = 18). Then, rats of each group were equally divided into three subgroups receiving 7 d, 14 d, and 28 d tail suspension, respectively, and then sacrificed at different times for liquid protein chip analysis.

### 2.3. 5-Bromo-2′-deoxyuridine (BrdU) Labeling and Tissue Preparation

Rats sacrificed after 7 d, 14 d, and 28 d tail suspension were treated with three pulses of BrdU (100 mg/kg, i.p. Sigma-Aldrich) with an 8 h interval 24 h before killing. For differentiation assay, the rats received a BrdU (100 mg/kg) injection each day for 7 d and were sacrificed 21 d after TS treatment (Figure 1).

At different times, the rats were deeply anesthetized with sodium pentobarbital (60 mg/kg, i.p.) and perfused transcardially with 0.9% saline, followed by 4% paraformaldehyde (pH 7.4). After cryoprotection with 30% sucrose in 0.01 M phosphate buffer saline (PBS), the brains were cut into 30 *μ*m coronal floating sections through the hippocampus containing the entire dentate gyrus (from bregma −2.64 mm to −5.64 mm) using a cryostat (Leica CM 1900). These free-floating sections were processed for immunostaining as described below. For BrdU immunohistochemistry, the sections were first incubated in 2 M HCl at 37°C for 30 min and neutralized with 0.1 M borate buffer (pH 8.5) for 10 min.

### 2.4. Immunohistochemistry

For immunohistochemistry, according to our previous study [18], floating sections were incubated with rat anti-BrdU (1 : 300, Abcam) or goat anti-doublecortin (DCX) (1 : 200, Abcam) or rabbit anti-ionized calcium binding adaptor molecule 1 (Iba1) IgG (1 : 500, Abcam) or rabbit anti-glial fibrillary acidic protein (GFAP) IgG (1 : 500, Abcam) in PBS containing 1% BSA and 0.3% Trition-X100 at 4°C overnight and then incubated with biotinylated anti-rat/goat/rabbit IgG (1 : 300, Vector) for 2 h at room temperature. For visualization of BrdU staining, the sections were incubated with avidin-biotin-peroxidase complex (1 : 300, Vector) for 2 h and then the brown color indicative of peroxidase activity was developed by incubating with 0.1% 3,3-diaminobenzidine (Sigma-Aldrich) and 0.05% H_2_O_2_ in PBS for 5 min at room temperature. For visualization of BrdU/DCX/Iba1/GFAP staining, the sections were incubated with cyanine2/cyanine3-conjuncted streptavidin (1 : 400; Jackson ImmunoResearch) for 2 h at room temperature. For double immunostaining, rat anti-BrdU (1 : 300, Abcam) and mouse anti-neuron-specific nuclear protein (NeuN) (1 : 200, Abcam) were added together at 4°C overnight, followed by Alexa Fluor-conjugated anti-rat IgG (1 : 500; Gibco/Invitrogen) and fluorescein isothiocyanate conjugated anti-mouse IgG (1 : 500; Sigma) for 2 h at room temperature. The specificity of immunolabeling was verified by negative controls in which the primary antibody was omitted (data not shown). Sections were observed under a Leica DMIRB or a Olympus FV-1000 confocal microscope.

### 2.5. Liquid Protein Chip Analysis

We subsequently examined the components of the hippocampus of each rat using a Luminex-based assay to detect which factors might be responsible for the activation of microglia. Rats were killed 1 d after weightlessness. The hippocampus of each rat was dissected and then homogenized in radioimmunoprecipitation assay (RIPA) buffer supplemented with phosphatase inhibitor and protease inhibitor. Lysates were centrifuged at 13,000 rpm for 30 min at 4°C. Protein concentration was determined by bicinchoninic acid protein assay. Then, the expressions of various cytokines, including IL-1*α*, IL-1*β*, IL-6, IL-10, IL-18, TNF-*α*, and IFN-*γ*, were detected in each rat using a Rat Cytokine/Chemokine Magnetic Bead Panel Kit (Millipore), as per manufacturer's instructions. In brief, 50 *μ*l of aliquots of standards or samples was incubated in a 1.2 *μ*m filter membrane 96-well microtiter plate with multicytokine beads for 2 h in the dark, washed using a vacuum manifold, and incubated with biotinylated reporter for 1.5 h. The plate was incubated with streptavidin–phycoerythrin for 30 min. After the final wash, the beads were evaluated using a Luminex 200 instrument and data collection and analysis was performed using the Milliplex Analyst software (Millipore). A minimum of 50 beads were analyzed.

### 2.6. Quantification and Statistical Analysis

For the quantification of BrdU^+^, Iba1^+^, and BrdU^+^/NeuN^+^ cells, the immunolabeled cells in the dentate gyrus of each brain were counted under a light microscope at 200x, and the results were expressed as the number of immunolabeled cells per dentate gyrus. To quantitatively analyze DCX and GFAP immunoreactivity, the staining intensity of immunoreactive structures evaluated by optical density (OD) was measured by the ImageJ software (National Institutes of Health). The background OD was taken from areas adjacent to the measured area. After the background density was subtracted, a ratio of the OD of image file was calibrated as % (relative OD, ROD). Finally, the ROD of DCX and GFAP immunoreactivity in the experimental group was normalized by dividing the ROD value of the control. At least three to five independent brains were included and six coronal sections (about 300 *μ*m apart) of each brain were used for counting or analysis.

All data were presented as mean ± standard deviation and analyzed using SPSS software for windows (version 20.0, IBM Corp, Armonk, NY, USA). Differences between groups were assessed using one-way ANOVA followed by Bonferroni post hoc test. A value of *p* < 0.05 was considered statistically significant.

## 3. Results

### 3.1. Microgravity Decreased Proliferation but Had No Effect on the Migration of NSCs in the Hippocampus

**Figure 2:**
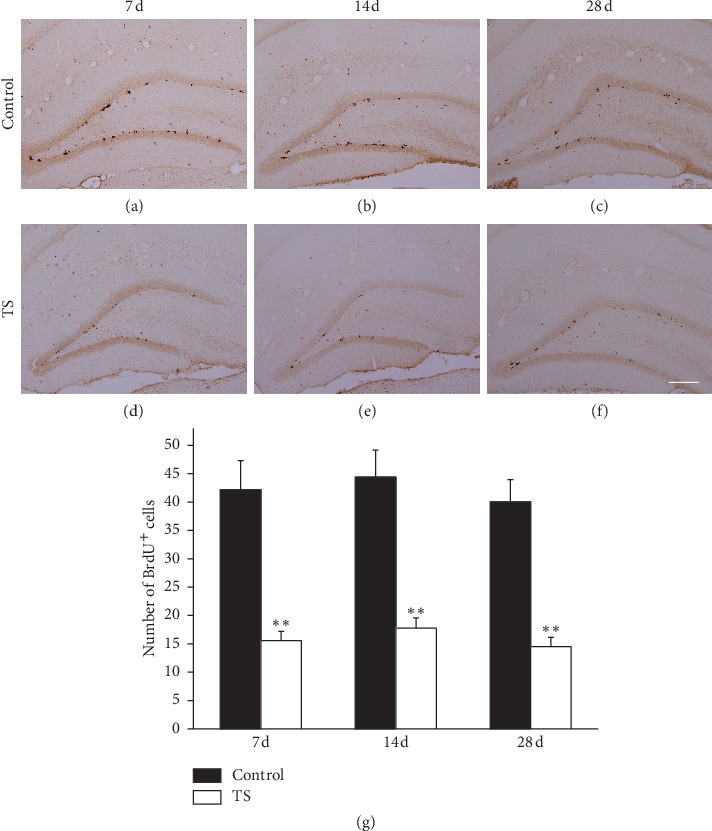
Microgravity suppressed the NSCs in the hippocampal dentate gyrus. Cell proliferation was assessed by BrdU labeling. Representative microphotographs show BrdU^+^ cells in the dentate gyrus of rats in control groups (a–c) and TS groups (d–f) which received 7, 14, and 28 d tail suspension, respectively. Scale bar: 200 mm. (g) Quantification of BrdU-positive cells in the dentate gyrus showing that, relative to the control, the number of BrdU^+^ cells was significantly decreased after 7, 14, and 28 d tail suspension. Error bars represent standard deviation (SD). ^*∗∗*^*P* < 0.01, compared with the control group (Bonferroni post hoc test after one-way ANOVA).

To investigate the effect of microgravity on NSC proliferation in the dentate gyrus, BrdU incorporation analysis was performed after three-pulse injection. In the TS groups, compared with the control group (Figures 2(a)–2(c)), the numbers of BrdU^+^ cells significantly decreased in the dentate gyrus after 7 d, 14 d, and 28 d tail suspension (Figures 2(d)–2(f)). However, there was no significant discrepancy in TS groups at different time points. Corresponding quantitative data also showed a similar result (Figure 2(g)).

**Figure 3:**
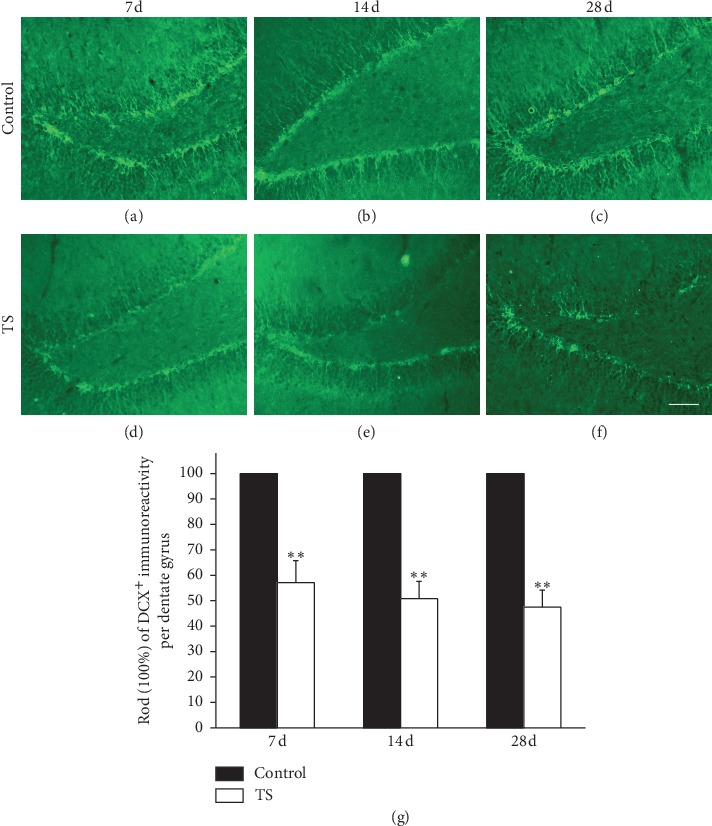
Microgravity decreased the number of DCX-labeled neural progenitors in the hippocampal dentate gyrus. Representative microphotographs showed DCX^+^ cells in the dentate gyrus of rats in control groups (a–c) and TS groups (d–f) received 7, 14, and 28 d tail suspension, respectively. Scale bar: 50 mm. (g) ROD of DCX immunoreactivity in the dentate gyrus. At the time points of 7, 14, and 28 d, the ROD of TS groups was much higher than that of the control. Error bars represent SD. ^*∗∗*^*P* < 0.01, compared with control group (Bonferroni post hoc test after one-way ANOVA).

Additionally, the expression of DCX, an immature neuronal marker, downregulated after 7 d, 14 d, and 28 day tail suspension (Figure 3). Besides, as illustrated in Figure 3, most DCX-positive neurons in the control group had their soma located in either the SGZ or the inner one-third of the granule cell layer, with a significant dendritic growth into the outer two-thirds of the dentate molecular layer. Furthermore, the location of DCX-positive neurons and the direction of their dendrite did not change at different time points, which indicate that microgravity had no effect on the migration of NSCs in the hippocampus.

### 3.2. Microgravity Did Not Affect NSC Differentiation in the Hippocampus

**Figure 4:**
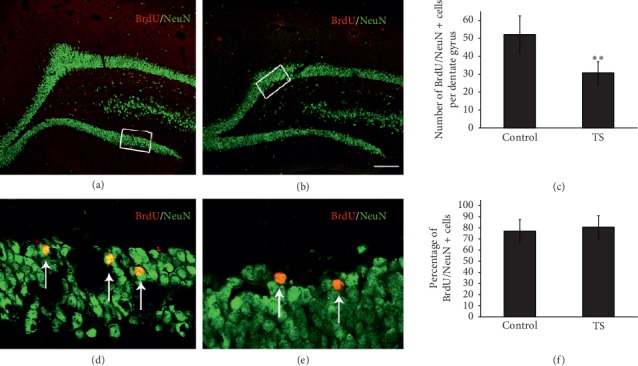
Microgravity does not affect the differentiation of NSCs in the hippocampal dentate gyrus. (a, b) Representative confocal microscope images show NeuN (green) and BrdU (red) double-labeled cells in the dentate gyrus at four weeks after BrdU injection. (d, e) The high-magnification views of the rectangular areas in their respective panels. The arrows in (d) and (e) show BrdU and NeuN double-labeled cells. Scale bars: 200 mm (a, b); 25 mm (d, e). (c) Quantification of BrdU^+^/NeuN^+^ cells in the dentate gyrus. In comparison with the control, exposure to microgravity significantly decreased the number of BrdU^+^/NeuN^+^ cells. (f) The percentage of BrdU^+^/NeuN^+^ cells to the total BrdU + cells did not differ between the control and TS groups. Error bars represent SD. ^*∗∗*^*P* < 0.01, compared with the control (Bonferroni post hoc test after one-way ANOVA).

In order to investigate the effect of microgravity on NSC differentiation, double labeling of BrdU and NeuN was performed 21 d after the last pulse injection of BrdU. In the TS group (Figures 4(b)–4(e)), BrdU/NeuN double-labeled cells were significantly decreased in the dentate gyrus compared with the control (Figures 4(a)–4(d)). Corresponding quantitative data also showed a similar result (Figure 4(c)). However, when examining the proportion of double-labeled cells in overall BrdU^+^ cells, we found that the ratio of BrdU^+^/NeuN^+^ to the total number of BrdU^+^ cells (about 80%) did not differ between the TS group and the control (Figure 4(f)). These results suggest that microgravity did not affect NSC differentiation.

Besides, owing to the low ratio of NSCs differentiating into astrocytes, rare BrdU/GFAP double-labeled cells were detected in the hippocampus of rats in either the control group or TS group (data not shown).

### 3.3. Minocycline Attenuated Microgravity-Induced Proliferation Reduction of NSCs

**Figure 5:**
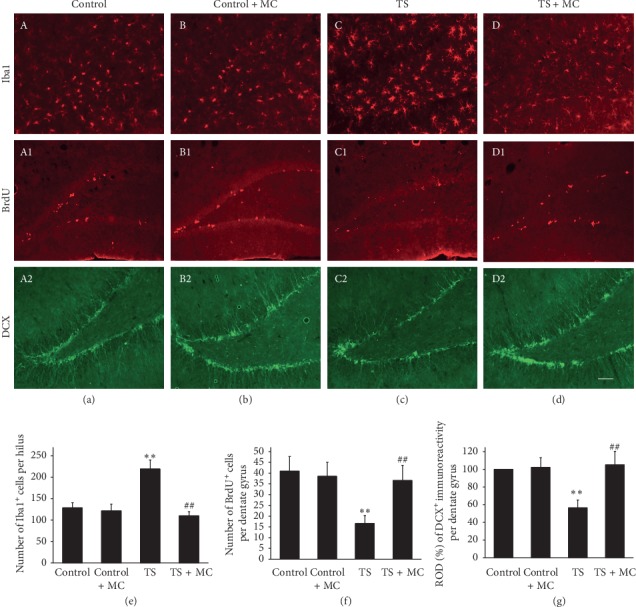
Minocycline suppressed the activation of microglia and the reduction in the number of NSCs in the dentate gyrus induced by microgravity. Representative microphotographs show that Iba1^+^, BrdU^+^, and DCX^+^ cells in the dentate gyrus of rats in control (a), control + MC (b), TS (c), and TS + MC groups (d) received 7-day tail suspension, respectively. (e) Quantification of the ROD of Iba1 immunoreactivity in the dentate gyrus. (f) Quantification of BrdU-positive cells in the dentate gyrus. (g) Quantification of the ROD of DCX immunoreactivity in the dentate gyrus. The ROD of Iba1 immunoreactivity in TS groups was much higher than that in the control; however, the increase was restrained by minocycline treatment. The number of BrdU^+^ cells and the ROD of DCX immunoreactivity of TS groups were much lower than those of the control; however, the decreases were reversed by minocycline treatment. Scale bar: 100 mm. Error bars represent SD. ^*∗∗*^*P* < 0.01, compared with control group; ^##^*P* < 0.01, compared with TS group (Bonferroni post hoc test after one-way ANOVA).

We next tested whether microglia affected the microgravity-induced reduction of the number of NSCs in the hippocampus. As expected, microglia were activated (Figure 5C) and the number of NSCs was reduced (Figure 5C1 and C2) remarkably after 7 d tail suspension, whereas MC administration significantly inhibited the Iba1 immunoreactivity in the dentate hilus (Figure 5D) and meanwhile reversed the reduction in the number of NSCs after exposure to microgravity, which was reflected by the increase in the number of BrdU^+^ cells (Figure 5D1) and in DCX immunoreactivity (Figure 5D2) in the SGZ. Quantitative analysis revealed the same result (Figures 5(e)–5(g)).

### 3.4. Microgravity Promoted the Concentrations of INF-*γ* and TNF-*α* in the Hippocampus

**Figure 6:**
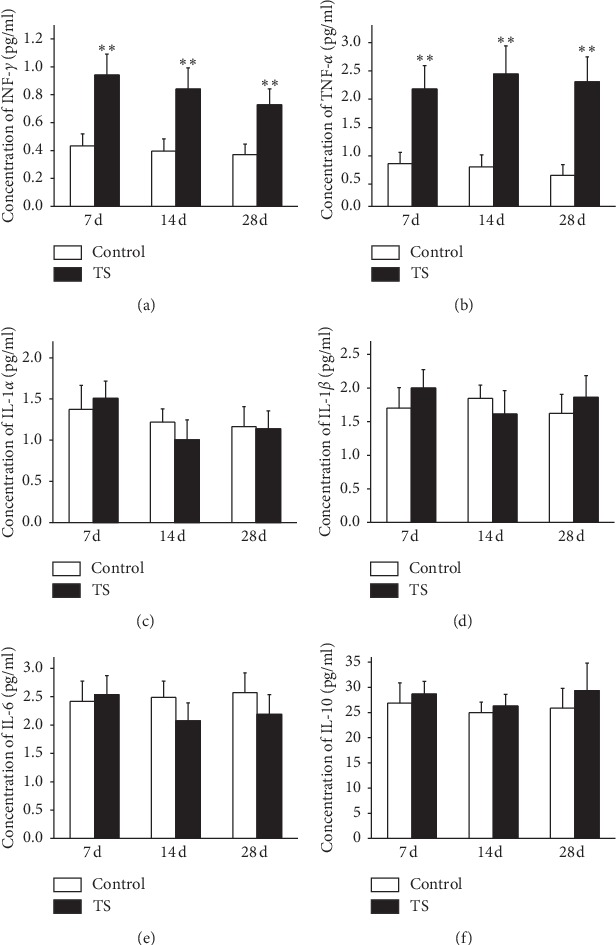
Microgravity changed the concentration of some inflammatory cytokines in rat hippocampus. The levels of INF-*γ* (a), TNF-*α* (b), IL-1*α* (c), IL-1*β* (d), IL-6 (e), and IL-10 (f) in supernatants of hippocampal tissue collected after 7-, 14-, or 28-day tail suspension were observed separately. Compared with control groups, the concentrations of INF-*γ* and TNF-*α* were much higher in TS groups at different time points. However, the concentration of other factors, such as IL-1*α*, IL-1*β*, IL-6, and IL-10, did not change much between the control and TS groups. ^*∗∗*^*P* < 0.01 versus control (Bonferroni post hoc test after one-way ANOVA).

Subsequently, we examined the components of the hippocampus of each rat using a Luminex-based assay to detect which factors might be responsible for the activation of microglia. Compared with the control group, rat hippocampi in the TS group contained significantly higher levels of proinflammatory cytokines INF-*γ* (Figure 6(a)) and TNF-*α* (Figure 6(b)) after 7, 14, and 28 d tail suspension. However, the concentrations of INF-*γ* and TNF-*α* did not have statistically significant differences in different time points. On the other hand, the levels of other cytokines, including IL-1*α*, IL-1*β*, IL-6, and IL-10, did not change following tail suspension (Figures 6(c)–6(f)).

## 4. Discussion

Microgravity, as a basic environmental factor in spaceflight, can influence physiological conditions as well as psychological functions. Recently, a series of studies evaluated cognitive function during space travel or parabolic flights, some of which proved that cognitive function shows a certain degree of decline during exposure to microgravity [4, 19–21]. As hippocampal neurogenesis is closely related to animals' ability to learn and memorize, in the present study, we firstly demonstrated that microgravity inhibited the proliferation of adult hippocampal NSCs in rats, which may tentatively explain the detrimental effects of microgravity on learning and memory.

In addition to proliferation, differentiating into newborn neurons is another important process during neurogenesis. The present study revealed that microgravity seemingly decreased the number of BrdU/NeuN double-labeled newborn neurons; however, the ratio of BrdU^+^/NeuN ^+^ cells to the total number of BrdU ^+^ cells did not differ from the control. These results suggest that the decrease in the number of newborn neurons may be the result of microgravity-induced attenuation of NSC proliferation, and microgravity itself does not affect the differentiation of NSCs. Our result is the first report of the differentiation of NSCs after microgravity. In a few previous studies, microgravity was found to enhance the differentiation of mesenchymal stem cells into neurons [22, 23]. This discrepancy may be explained by different stem cell types or the experimental method.

Adult neurogenesis can be regulated by various endogenous and exogenous modulators, and microgravity is likely to be involved in regulating the expression of these factors. Microglia are small cells present in the CNS and exhibit multiple biofunctions via synergizing with other neural cells. When changes occur in healthy brains in response to damage, microglia are immediately activated by danger signals and undergo great changes in morphology and function that include enlargement and thickening processes, proinflammatory protein production, and behavioral changes in proliferation, migration, and phagocytosis [24, 25]. Studies show that microglia are involved in each step of neurogenesis after different CNS damage. For example, microglia produce inflammatory cytokines to reduce the survival of newborn neurons after focal cerebral ischemia [26]. Inhibiting microglia activation improves spatial memory and adult neurogenesis in the rat hippocampus during 48 h of sleep deprivation [27]. Next, we investigated the function of microglia in neurogenesis after exposure to microgravity and found that the microglia were activated and the number of NSCs decreased strikingly in rat hippocampus after 7 d tail suspension. However, by using minocycline to inhibit the activation of microglia, the number of NSCs in the hippocampus returned to the normal level.

Excessive activation of microglia can exacerbate inflammatory responses in the CNS. Inflammatory responses play a crucial role in CNS damage and are regarded as an early phenomenon in the CNS damage process [28]. Our present findings demonstrated that higher concentrations of proinflammatory cytokines, including INF-*γ* and TNF-*α*, were detected in rat hippocampi following exposure to microgravity, which might be associated closely with microglial activation. The roles of INF-*γ* and TNF-*α* secreted by microglia have been reported in other CNS neurodegenerative diseases. The latest study suggests that chronic unpredictable mild stress increases microglia M1 marker CD11b expression and concentrations of TNF-*α*, INF-*γ*, IL-1*β*, and IL-17 [29]. Other researches prove that proinflammatory cytokines such as IL-1b, IL-6, and TNF-a are released by microglia and upregulate after ischemic stroke [30, 31].

Current evidence illustrates that the cytokines TNF-*α* and INF-*γ*, secreted by microglia, have been proven to be able to induce a reduction in the number of NSCs, and the possible mechanism has been investigated [32, 33]. For example, TNF-*α* induces NSCs apoptosis by activating the p38 MAPK signaling pathway [32]. IFN-*γ* could contribute to a reduction in NSC proliferation in inflammatory conditions through the activation of signal transducers and activators of transcription (STAT)1 and modulation of retinoblastoma protein phosphorylation [33]. These research studies represent potential gene targeting that may prevent unnecessary NSC loss in CNS infections and other inflammatory events disrupting brain neurogenesis.

Based on the results above, we speculate that microglia activate following exposure to microgravity and decrease NSC proliferation in the hippocampus, which may be related to the markedly increased concentrations of TNF-*α* and IFN-*γ*. However, the underlying mechanism is still unclear. Our next work will seek to explore the effects of TNF-*α* and IFN-*γ* on the proliferation, differentiation, and survival of NSCs after exposure to microgravity in vitro in order to provide more sufficient evidences. Based on this, we will try our best to reveal the molecular mechanism of proinflammatory factors on neurogenesis. Nevertheless, our studies provide a new idea that pharmacological blockade of TNF-*α* and IFN-*γ* function may offer a potent therapy for microgravity-elicited learning and memory impairment.

In addition to learning and memory impairment, reduced hippocampal neurogenesis also has great relation to depression. Previous studies indicate that adult hippocampal neurogenesis may underlie symptoms of psychiatric disorders, particularly depression [34–36], supported by recent studies indicating decreased numbers of granule cells and decreased granule cell layer volume in the dentate gyrus in depressed patients relative to controls, as well as increased hippocampal neurogenesis and increased granule cell layer volume in depressed patients who had taken antidepressants relative to unmedicated patients [37–39]. Depression is one of the most frequently occurring psychiatric disorders during space flight. Simulated microgravity, mimicked by tail-suspended hind limbs in rats, can trigger a series of depressive symptoms similar to those observed in humans [40, 41]. The etiology of depression is complex. Recent studies reveal that impairment of the normal structure and function of microglia can lead to depression and associated impairments in neurogenesis [42], and the plasma levels of proinflammatory cytokines, secreted by activated microglia, are correlated with depressive symptomatology [43, 44]. The microglial inhibitory drug minocycline has been identified as a potential novel treatment for depression [45]. Significant improvement in both depressive and psychotic symptoms in adult in patients with psychotic depression is found after 6 weeks of minocycline treatment [46]. However, conflicting findings from other research show that inhibition of microglial activation by minocycline does not necessary elicit antidepressant effects [47]. Our present study identified the reduction of neurogenesis after microgravity and raised the possible mechanism, inflammatory reaction mediated by microglia. Whether minocycline can exert antidepression effect after microgravity will be an interesting research direction in our future studies.

## 5. Novelty

Our research was the first study revealing that microgravity significantly suppressed the proliferation of NSCs in the hippocampus of rats but did not affect the migration and differentiation of them. Based on that, we investigated the possible mechanism underlying for the first time and found that microglia in hippocampus were activated remarkably following microgravity. After treated with minocycline, activated microglia were suppressed and the reduction of NSCs induced by microgravity recovered. Besides, compared with the control, higher concentrations of INF-*γ* and TNF-*α* were detected in the rats treated with microgravity.

## Data Availability

The data used to support the findings of this study are available from the corresponding author upon request.
